# Sub-exponential Time Parameterized Algorithms for Graph Layout Problems on Digraphs with Bounded Independence Number

**DOI:** 10.1007/s00453-022-01093-w

**Published:** 2023-01-11

**Authors:** Pranabendu Misra, Saket Saurabh, Roohani Sharma, Meirav Zehavi

**Affiliations:** 1 Chennai Mathematical Institute, Chennai, India; 2 Institute of Mathematical Sciences, HBNI, Chennai, India; 3 Max Planck Institute for Informatics, Saarland Informatics Campus, Saarbrücken, Germany; 4 Ben-Gurion University, Beersheba, Israel

**Keywords:** Sub-exponential fixed-parameter tractable algorithms, Directed feedback arc set, Directed cutwidth, Optimal linear arrangement, Bounded independence number digraph, 05C20, 05C85, 68Q25, 68R05, 68W40, 97K20, 97P20

## Abstract

Fradkin and Seymour (J Comb Theory Ser B 110:19–46, 2015) defined the class of digraphs of bounded independence number as a generalization of the class of tournaments. They argued that the class of digraphs of bounded independence number is structured enough to be exploited algorithmically. In this paper, we further strengthen this belief by showing that several cut problems that admit sub-exponential time parameterized algorithms (a trait uncommon to parameterized algorithms) on tournaments, including Directed Feedback Arc Set, Directed Cutwidth and Optimal Linear Arrangement, also admit such algorithms on digraphs of bounded independence number. Towards this, we rely on the generic approach of Fomin and Pilipczuk (in: Proceedings of the Algorithms—ESA 2013—21st Annual European Symposium, Sophia Antipolis, France, September 2–4, 2013, pp. 505–516, 2013), where to get the desired algorithms, it is enough to bound the number of *k*-cuts in digraphs of bounded independence number by a sub-exponential FPT function (Fomin and Pilipczuk bounded the number of *k*-cuts in transitive tournaments). Specifically, our main technical contribution is a combinatorial result that proves that the yes-instances of the problems (defined above) have a sub-exponential number of *k*-cuts. We prove this bound by using a combination of chromatic coding, inductive reasoning and exploiting the structural properties of these digraphs.

## Introduction

Tournaments form one of the most well studied families of digraphs, both algorithmically and structurally. In particular, whenever we try to generalize results that hold for undirected graphs to digraphs, arguably, one of the first families to consider is that of tournaments. Indeed, this has been the case when designing parameterized algorithms or approximation algorithms. Two problems that have been extensively studied on tournaments are Directed Feedback Vertex Set (DFVS) and Directed Feedback Arc Set (DFAS).

In the realm of approximation, we know that DFVS admits a 7/2-approximation algorithm on tournaments [20], and DFAS admits a PTAS on tournaments [18]. Here, it is worth to point out that whether or not DFAS is NP-complete on tournaments was a well known open problem in the area [4]. First, Ailon et al. [1] proved that unless NP$\subseteq$BPP, DFAS on tournaments admits no polynomial-time algorithm. Shortly afterwards, the proof that DFAS is NP-complete was attained simultaneously and independently by Alon [2] and Charbit et al. [6].

For DFVS on tournaments, the best known parameterized algorithm runs in time $1.618^k\cdot n^{\mathcal {O}(1)}$ [19]. Prior to this the fastest known parameterized algorithm for DFVS ran in time $2^k\cdot n^{\mathcal {O}(1)}$ [10], based on iterative compression. As in the case of approximation, from the viewpoint of Parameterized Complexity, DFAS on tournaments is “easier” than DFVS on tournaments. Here, we mean that for DFAS on tournaments, sub-exponential time parameterized algorithms are known. The quest for sub-exponential time parameterized algorithms for DFAS has a rich history. For a long time (even after the $2^k\cdot n^{\mathcal {O}(1)}$-time algorithm for DFVS was discovered), the question of the existence of an algorithm for DFAS that runs in time $2^k\cdot n^{\mathcal {O}(1)}$ was still being posed as an open problem.

Based on a generic method called chromatic coding (also used in our paper), Alon et al. [3] gave the first sub-exponential time parameterized algorithm for DFAS, which runs in time $2^{\mathcal {O}(\sqrt{k}\log ^2k)}\cdot n^{\mathcal {O}(1)}$. This was the first problem not confined to planar graphs (or generalizations such as apex-minor-free graphs) that was shown to admit a sub-exponential time parameterized algorithm. Later, simultaneously and independently, Feige [11] and, Karpinski and Schudy [17] gave faster algorithms that run in time $2^{\mathcal {O}(\sqrt{k})}\cdot n^{\mathcal {O}(1)}$. Fomin and Pilipczuk [13] presented a general approach, based on a bound on the number of *k*-cuts (defined below) in transitive tournaments, that achieved the same running time for DFAS. Using this framework they also designed the first sub-exponential time algorithms for Directed Cutwidth and Optimal Linear Arrangement (OLA) (defined later) on tournaments. Barbero et al. [5] studied Directed Cutwidth and OLA on semi-complete digraphs (that is, digraphs where for any two vertices *u* and *v*, at least one of the arcs (*u*, *v*) and (*v*, *u*) is present) and showed that these problems are NP-complete on semi-complete digraphs. Furthermore, they showed that Directed Cutwidth does not a admit polynomial kernel on semi-complete digraphs but admits a polynomial Turing kernel. Finally, they obtained a linear vertex kernel for OLA on semi-complete digraphs.

The measure of directed cutwidth plays a key role in the work of Chudnovsky and Seymour [8] where it is shown that tournaments are well-quasi-ordered under immersion. This measure was considered by Chudnovsky et al. [7] also in their algorithmic study of Immersion on tournaments. Later, Fradkin and Seymour [14] showed that the Directed Pathwidth and Topological Containment problems on tournaments are fixed parameter tractable (FPT). Fomin and Pilipczuk [12, 13], and Pilipczuk [21] revisited these problems and gave the best known algorithms for them on tournaments. Fradkin and Seymour [15], to generalize their results from tournaments to broader families of graphs, introduced the idea of digraphs that have bounded independence number. In particular, tournaments have independence number 1. They showed that Edge disjoint Paths admits an XP algorithm (that is, an algorithm with running time of the form of $n^{f(k)}$, where *n* is the number of vertices in the input graph and *k* is the number of pairs between which one is asked to find edge-disjoint paths) on this family of graphs.

In this paper, we study well-known cut problems (DFAS, Directed Cutwidth and OLA) on digraphs of bounded independence number. Our main contribution is proving a sub-exponential FPT bound on the number of *k*-cuts (defined below) in the Yes instances of these problems, which shows that the sub-exponential behavior of these problems on tournaments generalizes to digraphs of bounded independence number.

### Problem Statements and Our Algorithms

For a simple digraph *D* (every pair of vertices has at most one arc), denote $n=\vert V(D) \vert$ and $m=\vert E(D) \vert$. Let us formally define the class of digraphs relevant to our work. Given a digraph *D*, a vertex subset $I\subseteq V(D)$ is called an *independent set* if there are no arcs between any pair of vertices in *I*. For any positive integer $\alpha$, let

$$\begin{aligned} \mathcal {D}_{\alpha } = \{D : \text{ maximum } \text{ independent } \text{ set } \text{ in } \text{ D } \text{ has } \text{ size } \text{ at } \text{ most } \alpha \}. \end{aligned}$$

Observe that for $\alpha =1$, ${\mathcal {D}}_\alpha$ is a family of tournaments. *For simplicity, we assume to work with simple digraphs. However, all our results hold also when the digraph is not simple. That is, for any pair of vertices u, v, both the arcs (u, v) and (v, u) can be present in the digraph.* A digraph is a *DAG* (Directed Acyclic Graph) if it has no directed cycles.

We first study the following problem.
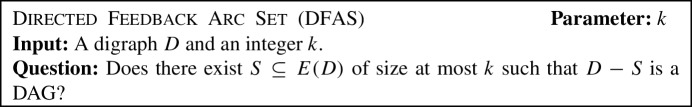


Our first theorem gives a sub-exponential time algorithm for DFAS on ${\mathcal {D}}_\alpha$.

#### Theorem 1.1

DFAS on ${\mathcal {D}}_\alpha$ is solvable in time $2^{\mathcal {O}(\alpha ^2 \sqrt{k} \log (\alpha k))} \cdot n^{\mathcal {O}(\alpha )}$.

Towards the definition of the second problem, let *D* be a digraph. For $X,Y \subseteq V(D)$, let $E(X,Y)= \{(u,v) \in E(D) : u \in X, v \in Y\}$ denote the set of arcs from *X* to *Y*. For an integer *q*, denote $[q]=\{1,\ldots ,q\}$. The *width* of an ordering $(v_1, \ldots , v_n)$ of *V*(*D*) is $\max _{i \in [n-1]} \vert E(\{v_{i+1}, \ldots , v_n\}, \{v_1,\ldots , v_i\})\vert .$ The *cutwidth* of *D*, denoted by ${\textbf {ctw}} (D)$, is the smallest possible width of an ordering of *V*(*D*). Now, the second problem is defined as follows.
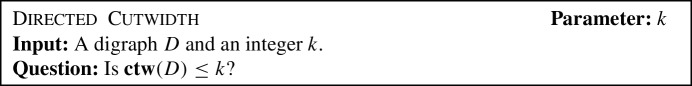


We present a sub-exponential time algorithm for Directed Cutwidth on ${\mathcal {D}}_\alpha$.

#### Theorem 1.2

Directed Cutwidth on ${\mathcal {D}}_\alpha$ is solvable in time $2^{\mathcal {O}(\alpha ^2 \sqrt{k} \log (\alpha k))} \cdot n^{\mathcal {O}(\alpha )}$.

Towards the definition of the third problem, let *D* be a digraph. For two integers *i*, *j*, let $[i >j]$ evaluate to 1 if $i >j$, and to 0 otherwise. The *cost* of an ordering $\sigma =(v_1, \ldots , v_n)$ of *V*(*D*) is $\sum _{(v_i,v_j) \in E(D)} (i-j) \cdot [i>j]$. In other words, every arc $(v_i,v_j)$ directed backward in $\sigma$ costs a value equal to its length, where the length of $(v_i,v_j)$ is the distance between $v_i$ and $v_j$ in $\sigma$. Our last problem seeks an ordering of cost at most *k*.
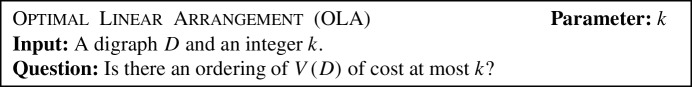


Our third theorem gives a sub-exponential time algorithm for OLA on ${\mathcal {D}}_\alpha$.

#### Theorem 1.3

OLA on ${\mathcal {D}}_\alpha$ is solvable in time $2^{\mathcal {O}(\alpha ^2 k^{\frac{1}{3}} \log (\alpha k))} \cdot n^{\mathcal {O}(\alpha )}$.

### Main Contribution and Methods

Our algorithms are based on the general framework of Fomin and Pilipczuk [13] to design parameterized sub-exponential time algorithms. The main ingredient to prove in order to employ this framework is a combinatorial upper bound on the number of “*k*-cuts” in graphs that are Yes-instances of the problem at hand. The proof for the combinatorial bound in our case is completely different from the proof given by Fomin and Pilipczuk [13] for transitive tournaments. The bound of Fomin and Pilipczuk [13] is achieved by mapping the set of *k*-cuts in a transitive tournament to the set of partitions of the integer *k*. Then, an asymptotic bound on the partition number of an integer yields a bound on the number of *k*-cuts in a transitive tournament. In the case of digraphs with bounded independence number, we do not know how to attain the desired bound by utilizing such partitions of integers.

Before we go further, we define the notion of *k*-cuts.

#### Definition 1

(*k*-*cut*) A *k*-*cut* in a digraph *D* is a partition of *V*(*D*) into two parts *L* and *R* (that is, $V(D) =L \uplus R$) such that $\vert E(R,L) \vert \le k$. The *k*-cut is denoted by the ordered pair (*L*, *R*). The set *L* is called the *left* part of the cut, and the set *R* is called the *right* part of the cut. The arcs in *E*(*R*, *L*) are the *cut-arcs* of (*L*, *R*).

Our first technical contribution is an upper bound on the number of *k*-cuts in ${\mathcal {D}}_\alpha$.

#### Lemma 1.4

If $D\in {\mathcal {D}}_\alpha$, then for any positive integer *k*, the number of *k*-cuts in *D* is at most $2^{c\sqrt{k} \log k} \cdot (n+1)^{2 \alpha \lceil \sqrt{k} \rceil } \cdot \log n$, where *c* is a fixed absolute constant.

The upper bound in Lemma 1.4 is of the form $n^{\mathcal {O}(f(\alpha )\sqrt{k})}$. That is, it shows that the number of *k*-cuts in digraphs in ${\mathcal {D}}_\alpha$ is upper bounded by a sub-exponential function in *n*. Clearly, such a bound is not sufficient to design sub-exponential time parameterized algorithms. If any of the problems DFAS, Directed Cutwidth or OLA on ${\mathcal {D}}_\alpha$ admits a polynomial kernel, then Lemma 1.4 can readily yield a sub-exponential time parameterized algorithm for the corresponding problem. However, we do not know whether these problems admit such kernels, and the resolution of these questions remains an interesting open problem.

Our second main technical contribution is an upper bound on the number of *k*-cuts in a *subfamily* of ${\mathcal {D}}_\alpha$. This bound suffices to prove Theorems 1.1, 1.2 and 1.3 by embedding it in the framework of Fomin and Pilipczuk [13]. Let us first define this subfamily. Given a vertex $v \in V(D)$, denote the set of out-neighbors of *v* in *D* by $N^{+}_D(v)=\{u \in V(D)~:~(v,u) \in E(D)\}$.

#### Definition 2

(*d*-*out-degenerate digraph*) For any positive integer *d*, a digraph *D* is *d*-*out-degenerate* if for every subgraph *H* of *D*, there exists a vertex $v \in V(H)$ such that $d^{+}_H(v) \le d$. An ordering $(v_1, \ldots , v_n)$ of the vertex set of *D* is a *d*-*out-degeneracy sequence* of *D* if for any $i \in \{2,\ldots ,n\}$, $\vert N(v_i) \cap \{v_j : j <i\}\vert \le d$.

Observe that a digraph is *d*-out degenerate if and only if it has a *d*-out-degeneracy sequence, that is there is an ordering of the vertex set of the digraph such that each vertex has at most *d* edges to the vertices before it. Also observe that DAGs are 0-out-degenerate. Next, we define a class of digraphs having small independence number and bounded out-degeneracy. Formally, $\mathcal {D}_{\alpha , d} =\{D :$ $D\in {\mathcal {D}}_\alpha$ and *D* is *d*-out-degenerate$\}$. Note that if $D \in \mathcal {D}_{\alpha ,d}$, then every induced subgraph $D'$ of *D* belongs to $\mathcal {D}_{\alpha ,d}$. Our second main technical contribution is formally stated as follows.

#### Lemma 1.5

If $D\in {{\mathcal {D}}}_{\alpha ,d}$, then for any positive integer *k*, the number of *k*-cuts in *D* is at most $2^{c(\alpha +1)\sqrt{k} \log k} \cdot (d+1+\alpha (2k+1))^{2 \alpha (\alpha +1) \lceil \sqrt{k} \rceil } \cdot \log (d + \alpha (2k+1)) \cdot n^{\alpha +1}$, where *c* is a fixed absolute constant.

One can easily see that if (*D*, *k*) is a Yes-instance of DFAS, Directed Cutwidth or OLA, then *D* is *k*-out-degenerate. Thus, Lemma 1.5 implies a sub-exponential (in *k*) upper bound on the number of *k*-cuts for Yes-instances of these problems. In fact, for OLA one can show that *D* is $2k^{2/3}$-out-degenerate, and thus obtain an improved upper bound on the number of *k*-cuts for Yes-instances. Since the *k*-cuts of any digraph can be enumerated with polynomial delay [13], hence the upper bounds in Lemmas 1.4 and 1.5 are constructive.

In what follows, we present our proof strategies for the results stated above.

*Proof Strategy of Lemma* 1.4 We first make a very simple observation, which serves as the starting point of our proof. Let $V(D) = V_1 \uplus \cdots \uplus V_{\ell }$ be some partition of *V*(*D*). Then, the number of *k*-cuts in *D* is upper bounded by the product of the number of *k*-cuts in the digraph induced by each $V_i$. Thus, we aim to partition *V*(*D*) into parts that induce “sufficiently structured” subdigraphs—we want the number of *k*-cuts in $D[V_i]$, for any $i \in [\ell ]$, to be “easier” to upper bound than the number of *k*-cuts in *D* directly. Moreover, since our aim is to achieve a bound of $n^{o(k)}$ for the total number of *k*-cuts in *D*, we want a partition $V(D) = V_1 \uplus \cdots \uplus V_{\ell }$ where $\ell = o(k)$. To this end, we utilize Gallai-Milgram’s Theorem (explained next) under the canvas of chromatic coding.

On the one hand, Gallai-Milgram’s Theorem states that if the size of a maximum independent set in a digraph is $\alpha$, then its vertex set can be partitioned into at most $\alpha$ parts such that the digraph induced by each of these parts has a directed Hamiltonian path. On the other hand, chromatic coding (in its derandomized form) provides a family $\mathcal {F}$ of partitions of *V*(*D*) such that *(i)* $\vert \mathcal {F}\vert = 2^{o(k)}\log n$, *(ii)* for each *k*-cut (*L*, *R*) in *D*, there exists a partition $\mathcal {P} \in \mathcal {F}$ such that all the cut arcs of (*L*, *R*) go across the parts of $\mathcal {P}$, and *(iii)* the number of parts of each partition in $\mathcal {F}$ is upper bounded by $\mathcal {O}(\sqrt{k})$. If the cut-arcs of (*L*, *R*) go across the parts of a partition $\mathcal {P}$, we say that (*L*, *R*) *respects* $\mathcal {P}$. To see how to combine these two tools, let $\mathcal {F}$ be a family provided by chromatic coding. Since the number of partitions in $\mathcal {F}$ is $2^{o(k)}\log n$, and for each *k*-cut (*L*, *R*) there exists a partition in $\mathcal {F}$ that it respects, it suffices to bound the number of *k*-cuts that respect a particular (arbitrary) partition in $\mathcal {F}$. Then, the total number of *k*-cuts in the digraph will be the product of the number of *k*-cuts that respect a partition in $\mathcal {F}$, over all partitions in $\mathcal {F}$.

Consider an arbitrary partition $\mathcal {P} \in \mathcal {F}$ (of *V*(*D*)). Let $\mathcal {P}=P_1 \uplus \cdots \uplus P_{\ell }$. Recall that $\ell = \mathcal {O}(\sqrt{k})$, and the number of *k*-cuts in *D* is at most the product of the number of *k*-cuts in $D[P_i]$, over all $i \in [\ell ]$. Here, a crucial insight is that the number of *k*-cuts in *D* that respect $\mathcal {P}$ is at most the product of the number of 0-cuts in $D[P_i]$, over all $i \in [\ell ]$. Thus, we have reduced our problem to upper bounding the number of 0-cuts in a digraph. Now, to upper bound the number of 0-cuts in $D[P_i]$ by $n^{o(k)}$, we utilize Gallai-Milgram’s Theorem. Since $D[P_i] \in \mathcal {D}_{\alpha }$, Gallai-Milgram’s Theorem implies that $P_i$ can be partitioned into at most $\alpha$ parts, say $P_i = P_{i1} \uplus \ldots \uplus P_{iq}$, $q \le \alpha$, such that for each $j \in [q]$, $D[P_{ij}]$ has a directed Hamiltonian path. Thus, we have finally reduced our problem to finding 0-cuts in digraphs that have a directed Hamiltonian path. As we will see later, the number of 0-cuts in such digraphs is linear in its number of vertices. Combining everything together, we are able to bound the number of *k*-cuts in *D* by $n^{\mathcal {O}(\alpha \sqrt{k})}$.

**Proof Strategy of Lemma** 1.5: Each vertex in a digraph *D* has two choices of how to participate in a cut—it can belong either to its left side or to its right side. Thus, if $|V(D)|=n$, a trivial upper bound on the total number of *k*-cuts in *D* is $2^n$. Suppose that we have (somehow) reached a “situation” where most of the vertices *must* belong to only one of the sides of a *k*-cut. Then, the arguments to attain the $2^n$ bound imply that the number of *k*-cuts is at most $2^{q}$, where *q* is the number of vertices which possibly have both choices. By the bound in Lemma 1.4, we can further conclude that the number of *k*-cuts is, in fact, at most $q^{\mathcal {O}(\alpha \sqrt{k})}$. Thus, if $q = k^{\mathcal {O}(1)}$ (that is, only $k^{\mathcal {O}(1)}$ vertices can choose a side), we get a bound of $2^{o(k)}$.

On a different note, suppose that we can identify a set of vertices in *D*, say $V_1$, such that $D[V_1]$ has at most $2^{o(k)}$ *k*-cuts. If $V_1$ is large enough, say $\vert V_1 \vert$ is such that $\vert V(D) {\setminus } V_1 \vert = k^{\mathcal {O}(1)}$, then we can bound the number of *k*-cuts in $D[V(D) {\setminus } V_1]$ by $2^{o(k)}$ (by Lemma 1.4). Since the number of *k*-cuts in *D* is bounded by the product of the number of *k*-cuts in $D[V_1]$ and the number of *k*-cuts in $D[V(D) {\setminus } V_1]$, we attain the bound of $2^{o(k)}$ on the number of *k*-cuts in *D*.

Our algorithm combines the two ideas above to obtain the desired bound. For any vertex $v\in V(D)$, we aim to bound the number of *k*-cuts in *D* where *v* is “forced” to belong to the left part. We exploit the position of *v* in a fixed *d*-out-degeneracy sequence of *D* to conclude that a large number of vertices are forced to belong to one side of these cuts. Then, building on the second idea, we inductively find a set of vertices such that the digraph induced on it has independence number strictly smaller than the independence number of *D*. For such a set of vertices, we can inductively assume that the number of *k*-cuts in the digraph induced by them is $2^{o(k)}$. Having this bound at hand, we are able to conclude the proof.

*Proof Strategy of Theorems* 1.1, 1.2 and 1.3 To obtain sub-exponential FPT algorithms for DFAS, Directed Cutwidth and OLA on ${\mathcal {D}}_\alpha$, we first use Lemma 1.5 to bound the number of *k*-cuts in the digraphs of the Yes-instances of these problems by $2^{o(k)}$. Here, we rely on the observation that these digraphs must be *k*-out-degenerate. Though we do not explicitly state this, the procedures to bound the number of *k*-cuts in both Lemmas 1.4 and 1.5 are constructive. However, constructiveness is not necessary since a standard branching procedure can also enumerate all *k*-cuts in a digraph with polynomial delay [13]. To actually solve any of the three problem, we rely on dynamic programming procedures over the *k*-cuts in the input digraph.

The last two steps of this proof (namely, the enumeration and the dynamic programming procedures) are quite standard, based on the work by Fomin and Pilipczuk [13] to obtain sub-exponential FPT algorithms for DFAS, Directed Cutwidth and OLA on tournaments. For the sake of completeness, we give the full proofs in this article too.

### Preliminaries

For any $i,j \in {\mathbb {Z}}^{+}$, denote $[i]=\{1, \ldots , i\}$, $[i]_0=\{0,1, \ldots , i\}$ and $[i,j]=\{i, i+1, \ldots , j-1 ,j\}$. For a partition $\mathcal {P} = P_1 \uplus \cdots \uplus P_{\ell }$, each $P_i$ is referred to as a *part* of $\mathcal {P}$. For a digraph *D*, *V*(*D*) denotes its vertex set and *E*(*D*) its arc set. We write $(u,v) \in E(D)$ if there is an arc in *D* with *u* as its tail and *v* as its head. Given a vertex $v \in V(D)$, the set of in-neighbors of *v* in *D*, denoted by $N^{-}_D(v)$, is the set of all vertices $u \in V(D)$ such that $(u,v) \in E(D)$. The set of out-neighbors of *v* in *D*, denoted by $N^{+}_D(v)$, is the set of all vertices $u \in V(D)$ such that $(v,u) \in E(D)$. The set of neighbors of *v*, denoted by $N_D(v)$, is the union of $N^{-}_D(v)$ and $N^{+}_D(v)$. For a set $X \subseteq V(D)$, we let $N^{-}_X(v)$ denote the set of in-neighbors of *v* in *X*, that is, $N^{-}_X(v) = N^{-}_D(v) \cap X$ (respectively, $N^{+}_X(v) = N^{+}_D(v) \cap X$, $N_X(v) = N_D(v) \cap X$). Whenever the digraph is clear from the context, we drop the subscript *D*. For $X,Y \subseteq V(D)$, $E(X,Y)= \{(u,v) : (u,v) \in E(D), u \in X \text { and } v \in Y\}$ denotes the set of arcs from *X* to *Y*. By *D*[*X*], we denote the directed subgraph induced by the vertices of *X*. A set $X \subseteq V(D)$ is called an *independent set* of *D* if for any $u,v \in X$, $(u,v) \not \in E(D)$ and $(v,u) \not \in E(D)$. In other words, *X* is an independent set in the underlying undirected graph of *D*. The independence number of a digraph is equal to the size of the maximum independent set it contains. A *directed Hamiltonian path* in *D* is a directed simple path on all vertices in *D*. For a set of vertices $\{v_1, \dots , v_n\}$, let $(v_1, \ldots ,v_n)$ denote the ordering where for any $i \in [n]$, $v_i$ is the *i*th vertex of the ordering.

## Bounding the Number of *k*-cuts for Digraphs in $\mathcal {D}_{\alpha }$

In this section, we prove that the number of *k*-cuts in any digraph on *n* vertices with bounded independence number is $n^{o(k)}$. In particular we prove Lemma 1.4. Let us recall that a *k*-cut in a directed graph *D* is a partition of the vertex set of *D* into two parts, $V(D) =L \uplus R$, such that $\vert E(R,L) \vert \le k$. Let us note that a 0-cut in a digraph *D* is a partition (*L*, *R*) of the vertex set *V*(*D*) such that there are no arcs from *R* to *L* in *D*.

At the heart of the proof of Lemma 1.4 is a simple observation that helps us focus on parts of the digraph for which bounding the number of *k*-cuts is easier. This simple observation is then exploited to its fullest using two main tools - (1) the Gallai-Milgram’s Theorem and (2) chromatic coding. Let us state them formally. We begin by stating this key observation, followed by formally defining both these ideas.

### Lemma 2.1

Let *D* be a digraph and $k \in {\mathbb {Z}}^{+}$. Let $V(D)= V_1 \uplus \ldots \uplus V_{q}$ be some partition of *V*(*D*). For any $i \in [q]$, let $N_i$ be the number of *k*-cuts in $D[V_i]$, then the number of *k*-cuts in *D* is at most $\prod _{i \in [q]} N_i$.

### Proof

To prove the lemma, observe that, it is enough to prove that for any *k*-cut (*L*, *R*) in *D*, there exists *k*-cuts $(L_i,R_i)$, for each $i \in [q]$, in $D[V_i]$, such that $L = \cup _{i \in [q]} L_i$ and $R= \cup _{i \in [q]} R_i$. To see this, for any $i \in [q]$, let $L_i = L \cap V_i$ and $R_i= R \cap V_i$. Observe that, each $(L_i,R_i)$ is a *k*-cut in $D[V_i]$, otherwise (*L*, *R*) is not a *k*-cut in *D*. $\square$

Thus, if we can partition the vertex set of *D* into *o*(*k*) parts such that it is “easier” to bound the number of *k*-cuts in each of these parts, then we are done. At a high level, we will first partition the vertex set of *D* using chromatic coding, and then further partition each part of this partition using Gallai-Milgram’s Theorem. We will then conclude by proving that the number of *k*-cuts in each of the sub-parts is linear in the number of vertices. We now state the Gallai-Milgram’s Theorem formally.

### Proposition 1

([16], Gallai-Milgram Theorem, 1960) For any $\alpha \in {\mathbb {Z}}^{+}$ and $D \in \mathcal {D}_{\alpha }$, there exists a partition of $V(D) = V_1 \uplus \ldots \uplus V_{q}$, such that $q \le \alpha$ and for each $i \in [q]$, $D[V_i]$ has a directed Hamiltonian path.

Next, we state the technique of chromatic coding in its derandomized form. To this end, we first define *universal* (*n*, *k*, *r*)*-coloring family* and then state the known results about the existence of such a families of bounded size. This result is called the chromatic coding lemma. For any graph *G*, a *proper vertex coloring* of *G* is a function $f: V(G) \rightarrow {\mathbb {Z}}^{+}$, such that for any $(u,v) \in E(G)$, $f(u) \ne f(v)$.

### Definition 3

([3], *Universal* (*n*, *k*, *r*)-*Coloring Family*) For integers *n*, *k* and *r*, a family $\mathcal {H}$ of functions from [*n*] to [*r*] is called a universal (*n*, *k*, *r*)-coloring family, if for any graph *G* on the vertex set [*n*] with at most *k* edges, there exists an $h \in \mathcal {H}$ which is a proper vertex coloring of *G*.

Observe that the above mentioned definition holds for digraphs too, where the notion of proper vertex coloring is defined on its underlying undirected graph.

### Proposition 2

([3], Chromatic Coding Lemma) For any $n,k \ge 1$, there exists a universal $(n,k, 2 \lceil \sqrt{k} \rceil )$-coloring family of size at most $2^{\mathcal {O}(\sqrt{k} \log k)} \cdot \log n$.

A formulation of the Chromatic Coding lemma, in the way that is useful to us, can be seen in the following corollary.

### Corollary 1

For any digraph *D* on *n* vertices, and an integer *k*, there exists a family $\mathcal {F}$ of partitions of *V*(*D*) into at most $2 \sqrt{k}$ parts, such that,

1. for any *k*-cut (*L*, *R*) in *D*, there exists a partition $\mathcal {P} =\{P_1, \ldots ,P_q\}$ in the family $\mathcal {F}$, such that for any cut-arc (*u*, *v*) of (*L*, *R*), there exists $i,j \in [q]$, $i \ne j$, such that $u \in P_i$ and $v \in P_j$, and
2. $$\vert \mathcal {F} \vert = 2^{\mathcal {O}(\sqrt{k} \log k)} \cdot \log n$$.

### Proof

Let $\mathcal {H}$ be a $(n,k,2 \lceil \sqrt{k} \rceil )$-universal coloring family from Proposition 2, of size at most $2^{\mathcal {O}(\sqrt{k} \log k)} \cdot \log n$. We construct a family $\mathcal {F}$ of partitions of *V*(*D*) from the family $\mathcal {H}$ as follows. For each $h \in \mathcal {H}$, there is a partition $\mathcal {P}_h=P_1 \uplus \cdots \uplus P_{2\lceil \sqrt{k} \rceil }$ in $\mathcal {F}$, where for any $i \in [2\lceil \sqrt{k} \rceil ]$, $P_i= h^{-1}(i)$. Here, if for a certain *i*, $P_i = \emptyset$, then we discard this part from the partition $\mathcal {P}_h$.

We will now show that $\mathcal {F}$ is indeed the family with the required properties. Since $\vert \mathcal {H} \vert = 2^{\mathcal {O}(\sqrt{k} \log k)} \cdot \log n$, clearly $\vert \mathcal {F} \vert = 2^{\mathcal {O}(\sqrt{k} \log k)} \cdot \log n$. Let (*L*, *R*) be some *k*-cut in *D*. Consider the digraph, say $D_{(L,R)}$, on the vertex set of *D* with only the cut-arcs of (*L*, *R*). Note that $\vert E(D_{(L,R)}) \vert \le k$. Thus, from the definition of $(n,k,2 \lceil \sqrt{k} \rceil )$-universal coloring family, there exists a function $h: V(D_{(L,R)}) \rightarrow [2 \lceil \sqrt{k} \rceil ]$ in $\mathcal {H}$, such that *h* is a proper vertex coloring of $D_{(L,R)}$. Consider the partition $\mathcal {P}_h \in \mathcal {F}$. Let $\mathcal {P}_h= P_1 \uplus \cdots \uplus P_{2 \lceil \sqrt{k} \rceil }$. Since *h* is a proper coloring of $D_{(L,R)}$ and all the cut-arcs of (*L*, *R*) are in $D_{(L,R)}$, for any cut-arc (*u*, *v*) of (*L*, *R*), $h(u) \ne h(v)$. Thus, if $h(u)=i$ and $h(v)=j$, $i \ne j$, then $u \in P_i$ and $v \in P_j$. $\square$

For the rest of this section, let $\mathcal {F}$ denote the family described in Corollary 1 for the digraph *D* and integer *k*. For any arc (*u*, *v*) of a digraph and a partition $\mathcal {P}=P_1 \uplus \cdots \uplus P_q$ of the vertex set of the digraph, we say that the arc (*u*, *v*) *goes across the parts of this partition* $\mathcal {P}$, *if* $u \in P_i$, $v \in P_j$ *and* $i \ne j$. *For any partition* $\mathcal {P}$ *of the vertex set of the digraph* *D*, *we say that a* *k-cut* (*L*, *R*) *in* *D* *respects* $\mathcal {P}$ if all the cut-arcs of (*L*, *R*) go across the parts of $\mathcal {P}$. The next lemma states that, the number of *k*-cuts in *D* is at most the sum of the number of *k*-cuts that respect a partition $\mathcal {P}$, over all partitions $\mathcal {P} \in \mathcal {F}$. Since $\vert \mathcal {F} \vert = 2^{o(k)}$, it is enough to bound the number of *k*-cuts that respect an arbitrary partition in $\mathcal {F}$ by $n^{o(k)}$. For the digraph *D*, an integer *k* and $\mathcal {P} \in \mathcal {F}$, let $N_{\mathcal {P}}$ be the number of *k*-cuts in *D* that respect $\mathcal {P}$.

### Lemma 2.2

The total number of *k*-cuts in *D* is at most $\sum _{\mathcal {P} \in \mathcal {F}} N_{\mathcal {P}}$.

### Proof

To prove the lemma, we need to prove that for any *k*-cut (*L*, *R*) in *D*, there exists $\mathcal {P} \in \mathcal {F}$ such that (*L*, *R*) respects $\mathcal {P}$. This follows from Corollary 1. $\square$

Henceforth, let us fix $\mathcal {P} =P_1 \uplus \cdots \uplus P_q$, $q \le 2 \lceil \sqrt{k} \rceil$, where $\mathcal {P}$ is an arbitrary partition in $\mathcal {F}$. We are now only interested in bounding the number of *k*-cuts in *D* that respect $\mathcal {P}$. It follows from Lemma 2.1, that to bound the total number of *k*-cuts in *D*, it is sufficient to bound the number of *k*-cuts in $D[P_i]$, for each $i \in [q]$. We now have the following lemma, that says something much stronger. To bound the number of *k*-cuts in *D* that respect $\mathcal {P}$, it is sufficient to bound the number of just the 0-cuts in $D[P_i]$, for all $i \in [q]$.

### Lemma 2.3

For any digraph *D*, let $\mathcal {P}= P_1 \uplus \ldots \uplus P_{q}$ be some partition of the vertex set of *D*. For any $i \in [q]$, let $N_i$ be the number of 0-cuts in $D[P_i]$. Then the number of *k*-cuts in *D* that respect $\mathcal {P}$ is at most $\prod _{i \in [q]} N_i$.

### Proof

Observe that to prove the lemma it is enough to prove that for any *k*-cut (*L*, *R*) of *D* that respects $\mathcal {P}$, there exists 0-cuts $(L_i,R_i)$ in $D[P_i]$, for each $i \in [q]$ such that $L = \cup _{i \in q}L_i$ and $R = \cup _{i \in [q]} R_i$. Let (*L*, *R*) be some *k*-cut in *D* that respects $\mathcal {P}$. For each $i \in [q]$, let $L_i = L \cap P_i$ and $R_i = R \cap P_i$. Observe that, for each $i \in [q]$, $(L_i,R_i)$ is a 0-cut in $D[P_i]$. Suppose not. Then there exists a cut-arc of $(L_i,R_i)$, say (*u*, *v*), such that $u,v \in P_i$ and $u\in R_i$, $v \in L_i$. Since $L = \cup _{i \in [q]} L_i$ and $R= \cup _{i \in [q]} R_i$, $u \in R$ and $v \in L$. This contradicts that (*L*, *R*) respects $\mathcal {P}$. $\square$

Thus, we have further narrowed down the class of *k*-cuts that we want to bound. More precisely, we are now interested in bounding the number of 0-cuts in $D[P_i]$, for any part $P_i$ of $\mathcal {P}$. Since $D \in \mathcal {D}_{\alpha }$, for any $P_i \in \mathcal {P}$, $D[P_i] \in \mathcal {D}_{\alpha }$. Thus, from Gallai-Milgram Theorem, the vertex set of $P_i$ can be partitioned into at most $\alpha$ parts, say $P_i = P_{i1} \uplus \cdots \uplus P_{i{\ell }}$, $\ell \le \alpha$, such that for each $j \in [\ell ]$, $D[P_{ij}]$ has a directed Hamiltonian path. We will now prove that for any digraph that has a directed Hamiltonian path, the number of 0-cuts in it are linear in the number of its vertices.

### Lemma 2.4

Let *D* be a digraph on *n* vertices that has a directed Hamiltonian path. Then the number of 0-cuts in *D* is at most $n+1$.

### Proof

Since *D* has a directed Hamiltonian path, let $\{v_1, \ldots , v_n\}$ be the vertex set of *D* such that for each $i \in [n-1]$, $(v_i , v_{i+1}) \in E(D)$. Consider any 0-cut (*L*, *R*) in *D*. Let *i* be the smallest integer such that $v_i \in R$. By the choice of *i*, for all $j <i$, $v_j \in L$. We now claim that, for all $j >i$, $v_j \in R$. Suppose not. Then there exist a $j > i$, such that $v_j \in L$. Since $j>i$, and $v_i$ appears before $v_j$ in the Hamiltonian path ordering. Thus, there is a directed path from $v_i$ to $v_j$ in *D*. Since $v_i \in R$ and $v_j \in L$, an arc of this directed path is a cut-arc for (*L*, *R*), which contradicts that (*L*, *R*) is a 0-cut.

Thus, for any $i \in [n]$, the number of 0-cuts in *D* where $v_i$ is the first vertex in the ordering $(v_1, \ldots , v_n)$ that belongs to the right part of these cuts is exactly 1. Since any cut in *D*, either does not contain any vertex in its right part (there is only one such cut) or contains some vertex, the total number of 0-cuts in *D* is at most $n+1$. $\square$

**Fig. 1:**
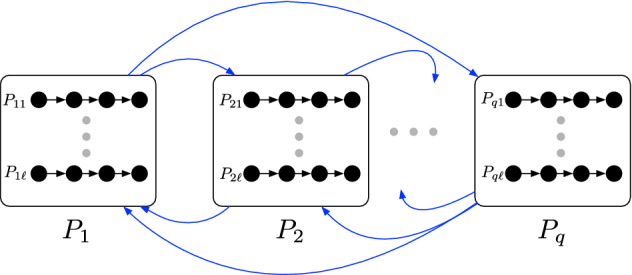
The vertex partition for the sub-exponential XP bound. $\mathcal {P} =\{P_1 \uplus \cdots \uplus P_q\}$ is the vertex partition obtained using chromatic coding and $P_i=P_{i1} \uplus \cdots \uplus P_{i\ell }$ is the partition obtained using Gallai–Milgram’s Theorem. Each $P_{ij}$ contains a Directed Hamiltonian Path. The cut arcs of all the cuts that respect $\mathcal {P}$ are marked in blue (Color figure online)

We are now ready to prove Lemma 1.4. An illustration depicting the partitioning used in the proof of Lemma 1.4 is given in Fig. 1.

### Proof of Lemma 1.4

Let *N* be the total number of *k*-cuts in *D*. Consider the family $\mathcal {F}$ of Corollary 1 for the digraph *D* and integer *k*. From Corollary 1, $\vert \mathcal {F} \vert \le 2^{\mathcal {O}(\sqrt{k} \log k)} \cdot \log n$. For each partition $\mathcal {P} \in \mathcal {F}$, let $N_\mathcal {P}$ be the number of *k*-cuts in *D* that respect $\mathcal {P}$. From Lemma 2.2, $N \le \sum _{\mathcal {P} \in \mathcal {F}} N_{\mathcal {P}}$. Consider any arbitrary partition $\mathcal {P} \in \mathcal {F}$. Let $\mathcal {P} = P_1 \uplus \ldots \uplus P_{q}$, and from Corollary 1 we have $q \le 2 \lceil \sqrt{k} \rceil$. For any $i \in [q]$, let $N_{P_i}$ be the number of 0-cuts in $D[P_i]$. From Lemma 2.3, $N_{\mathcal {P}} \le \prod _{i \in [q]} N_{P_i}$. Since $D \in \mathcal {D}_{\alpha }$, for any $P_i$, $D[P_i] \in \mathcal {D}_{\alpha }$. Thus, from Gallai–Milgram Theorem, the vertex set of $P_i$ can be partitioned into at most $\alpha$ parts, say $P_i = P_{i1} \uplus \ldots \uplus P_{i{\ell }}$, $\ell \le \alpha$, such that such that for each $j \in [\ell ]$, $D[P_{ij}]$ has a directed Hamiltonian path. From Lemma 2.4, the number of 0-cuts in $D[P_{ij}]$ is at most $n+1$. From Lemma 2.1, $N_{P_i} \le \prod _{j \in [\ell ]} (n_{i,j}+1) \le (n+1)^{\ell } \le (n+1)^{\alpha }$, where $n_{i,j}=|P_{i,j}|$. Combining everything stated above, we get that,

$$N \le \sum _{\mathcal {P} \in \mathcal {F}} N_{\mathcal {P}} \le \sum _{\mathcal {P} \in \mathcal {F}} \prod _{P_i \in \mathcal {P}}N_{P_i} \le \sum _{\mathcal {P} \in \mathcal {F}} \prod _{P_i \in \mathcal {P}}(n+1)^{\alpha } \le |\mathcal {F}| (n+1)^{2 \alpha \lceil \sqrt{k} \rceil } \le 2^{\mathcal {O}(\sqrt{k} \log k)} \cdot (n+1)^{2 \alpha \lceil \sqrt{k} \rceil } \cdot \log n$$. $$\square $$

## Improved Bounds for Digraphs in $\mathcal {D}_{\alpha }$ with Bounded Out-Degeneracy

In this section we give the proof of Lemma 1.5. Recall from the introduction that a digraph *D* is said to be *d*-out-degenerate, if for every subgraph *H* of *D*, there exists a vertex $v \in V(H)$, such that $d^{+}_H(v) \le d$. Furthermore, a digraph *D* *d*-out degenerate if and only if it has a *d*-out-degeneracy sequence.

Throughout this section, *D* is a digraph on *n* vertices and $D \in \mathcal {D}_{\alpha ,d}$. Let $(v_1, \ldots , v_n)$ be a *d*-out-degeneracy sequence of *D*. For any $i \in [n]$, we say that a *k*-cut (*L*, *R*) in *D* is of *type-i*, if $v_i \in L$ and for all $j >i$, $v_j \in R$. We say that a *k*-cut (*L*, *R*) in *D* is of *type-*0 if $L =\emptyset$. Note that the collection of the sets of type-*i* cuts for all $i \in [n]_0$, forms a partition of the set of all the *k*-cuts. Observe that there is exactly 1 type-0 cut in any digraph.

### Observation 1

For any $i \in [n]_0$, let $N_i$ be the number of *k*-cuts in *D* of type-*i*. Then the number of *k*-cuts in *D* is at most $\sum _{i \in [n]_0} N_i$.

Henceforth, our goal is to bound the number of *k*-cuts in *D* of type-*i*, for an arbitrary $i \in [n]$. Recall from Lemma 2.1 that if $V(D) = V_1 \uplus \cdots \uplus V_c$ is a partition of the vertex set of *D*, then to bound the number of *k*-cuts in *D*, it is enough to bound the number of *k*-cuts in each $D[V_j]$, $j \in [c]$. This remains our underlying strategy. However, this time we use a different partition of the vertex set of *D*, where the number of parts of this partition is 4, compared to *o*(*k*) in Lemma 1.4. This partition of the vertex set, is presented in Lemma 3.1.

### Lemma 3.1

For a digraph $D \in \mathcal {D}_{\alpha ,d}$ and any positive integer *k*, for any fixed $i \in [n]$, there exists a partition $V(D) = V_{\textrm{induct}} \uplus V_{\textrm{forceL}} \uplus V_{\textrm{forceR}} \uplus V_{\textrm{small}}$ such that:

1. If $\alpha =1$, then $V_{\textrm{induct}} =\emptyset$, otherwise $D[V_{\textrm{induct}}] \in \mathcal {D}_{\alpha ',d}$, where $\alpha ' <\alpha$.
2. For any *k*-cut (*L*, *R*) in *D* of type-*i*, $V_{\textrm{forceL}} \subseteq L$.
3. For any *k*-cut (*L*, *R*) in *D* of type-*i*, $V_{\textrm{forceR}} \subseteq R$.
4. $$\vert V_{\textrm{small}} \vert \le d+ \alpha (2k+1)$$.

Lemma 3.1 states that the vertex set of *D* can be partitioned into 4 parts with the following properties. The digraph induced on the *first* part is either empty or belongs to $\mathcal {D}_{\alpha ',d}$, for $\alpha ' < \alpha$. To bound the number of *k*-cuts in such a digraph we will use an induction on $\alpha$. For the *second* part of this partition, we prove that for any *k*-cut (*L*, *R*) of type-*i*, all the vertices of this part belong to *L*. Similarly, for the *third* part of this partition, we prove that for any *k*-cut (*L*, *R*) of type-*i*, all the vertices of this part belong to *R*. Therefore, there is a unique *k*-cut of type-*i* in the digraph induced by the second and third part. The *last* part of the partition has the property that the number of vertices in this part is “small”. For the digraph induced by this part, we will get the desired bound by using Lemma 1.4 on this digraph.

The proof of Lemma 3.1 is deferred for later. We will now proceed towards the proof of Lemma 1.5 using Lemma 3.1 and induction on $\alpha$. At any inductive step we use the partition of Lemma 3.1 and bound the number of *k*-cuts of type-*i* in the digraph induced on each part of the partition, thereby bounding the number of *k*-cuts in *D* because of Observation 1.

### Proof of Lemma 1.5

We prove the lemma using induction on $\alpha$. For any positive integer $\alpha$, let us denote the bound of Lemma 1.4 on the number of *k*-cuts in $D\in {\mathcal {D}}_\alpha$, on at most $d+\alpha (2k+1)$ vertices, by $\eta (\alpha ,d,k)$. That is, $\eta (\alpha ,d,k) = 2^{c\sqrt{k} \log k} \cdot (d+1+\alpha (2k+1))^{2 \alpha \lceil \sqrt{k} \rceil } \cdot \log (d+\alpha (2k+1))$, where *c* is the absolute constant hidden in the $\mathcal {O}$ notation of the expression in Proposition 2. Let $\mathcal {N}_k(n,\alpha ,d)$ denote the maximum number of *k*-cuts in *D* for any digraph $D \in \mathcal {D}_{\alpha ,d}$ on *n* vertices. We claim that for any positive integers *n*, *d* and $\alpha >1$, $\mathcal {N}_k(n,1,d) \le 1+ n \cdot \eta (1,d,k)$ and $\mathcal {N}_k(n,\alpha ,d) \le 1+ \mathcal {N}_k(n,\alpha -1,d) \cdot \eta (\alpha ,d,k) \cdot n$. Solving the recurrence, we will get the desired bound on the number of *k*-cuts in *D*.

Let us first prove that for any positive integers *n* and *d*, $\mathcal {N}_k(n,1,d) \le 1+ n \cdot \eta (1,d,k)$. If the independence number of the digraph *D* is 1, then from Lemma 3.1, there exists a partition $V(D)=V_{\textrm{forceL}} \uplus V_{\textrm{forceR}} \uplus V_{\textrm{small}}$ of *D* such that for any *k*-cut (*L*, *R*) in *D* of type-*i*, $V_{\textrm{forceL}} \subseteq L$ and $V_{\textrm{forceR}} \subseteq R$. Thus, from Lemma 2.1, we conclude that the number of *k*-cuts of type-*i* in *D* is at most the number of *k*-cuts in $D[V_{\textrm{small}}]$. Since $D[V_{\textrm{small}}]$ is an induced subgraph of *D*, the independence number of $D[V_{\textrm{small}}]$ is at most $\alpha$ and $D[V_{\textrm{small}}]$ is a digraph on $d + 2k + 1$ vertices. Thus, we conclude that the number of *k*-cuts in *D* of type-*i* is at most $\eta (1,d,k)$. From Observation 1, we conclude that the number of *k*-cuts in *D* is at most $1+ \eta (1,d,k) \cdot n$.

By induction hypothesis, let us assume that for any positive integers *n*, *d* and for all $\alpha '<\alpha$, the number of *k*-cuts in any digraph $D' \in \mathcal {D}_{\alpha ',d}$ on at most *n* vertices is $\mathcal {N}_k(n,\alpha ',d)$. We will now prove that the number of *k*-cuts in the digraph $D\in {{\mathcal {D}}}_{\alpha ,d}$ is $\mathcal {N}_k(n,\alpha ,d) \le 1+ \mathcal {N}_k(n,\alpha -1,d) \cdot \eta (\alpha ,d,k)$. From Lemma 3.1, there exists a partition $V(D) =V_{\textrm{induct}} \uplus V_{\textrm{forceL}} \uplus V_{\textrm{forceR}} \uplus V_{\textrm{small}}$, such that for any *k*-cut (*L*, *R*) in *D* of type-*i*, $V_{\textrm{forceL}} \subseteq L$ and $V_{\textrm{forceR}} \subseteq R$. Thus, from Lemma 2.1, the number of *k*-cuts of type-*i* in *D* is at most the product of the number of *k*-cuts in $D[V_{\textrm{induct}}]$ and the number of *k*-cuts in $D[V_{\textrm{small}}]$. Since $D[V_{\textrm{induct}}] \in \mathcal {D}_{\alpha ',d}$, where $\alpha ' < \alpha$, from inductive hypothesis we get that the number of *k*-cuts in $D[V_{\textrm{induct}}]$ is at most $\mathcal {N}_k(n,\alpha ',d) \le \mathcal {N}_k(n,\alpha -1,d)$. Since $\vert V_{\textrm{small}} \vert \le d+ \alpha (2k+1)$, from Lemma 1.4, the number of *k*-cuts in $D[V_{\textrm{small}}]$ is at most $\eta (\alpha ,d,k)$. Thus, the number of *k*-cuts of type-*i* in *D* is at most $\mathcal {N}_k(n,\alpha -1,d) \cdot \eta (\alpha ,d,k)$. From Observation 1, we conclude that the number of *k*-cuts in *D* is at most $1 +\mathcal {N}_k(n,\alpha -1,d) \cdot \eta (\alpha ,k,d) \cdot n$. $\square$

**Proof of Partitioning Lemma.** We start by a lemma that gives an upper bound on the size of a digraph in ${\mathcal {D}}_\alpha$ when every vertex has small out-degree.

### Lemma 3.2

For any digraph $D \in \mathcal {D}_{\alpha }$ and a positive integer *k*, if for all $v \in V(D)$, $d^{+}(v) \le k$, then $\vert V(D) \vert \le \alpha (2k+1)$.

### Proof

Let $|V(D)|=n$. We will first prove that if $D \in \mathcal {D}_{\alpha }$, then there exists $v \in V(D)$ such that $d^{+}(v) \ge \frac{(n- \alpha )}{2 \alpha }$. Since $d^{+}(v) \le k$, for all $v \in V(D)$, this implies that $\frac{(n - \alpha )}{2 \alpha } \le k$, thereby implying that $n \le \alpha (2k+1)$.

To prove the above-mentioned claim, we invoke Turan’s Theorem ( [9]), which states that for any graph *G* and integer *r*, if *G* does not contain a clique of size $r+1$, then $\vert E(G) \vert \le (1- \frac{1}{r}) \cdot \frac{\vert V(G) \vert ^2}{2}$. Let *G* be the underlying undirected graph of *D*. Let ${\bar{G}}$ be the complement graph of *G*. Since $D \in \mathcal {D}_{\alpha }$, ${\bar{G}}$ does not contain a clique of size $\alpha +1$. Thus, by Turan’s Theorem, $\vert E({\bar{G}}) \vert \le (1- \frac{1}{\alpha }) \cdot \frac{n^2}{2}$. Since ${\bar{G}}$ is the complement graph of *G*, $\vert E(G) \vert \ge \frac{n (n -1)}{2} - (1- \frac{1}{\alpha }) \cdot \frac{n^2}{2} \ge \frac{(n^2 - n \alpha )}{2 \alpha }$. Since *G* is the underlying undirected graph of *D*, $\vert E(D) \vert \ge \frac{(n^2 - n \alpha )}{2 \alpha }$. Since $\vert E(D) \vert = \sum _{v \in V(D)} d^{+}(v) \ge \frac{(n^2 - n\alpha )}{2 \alpha }$, there exists $v \in V(D)$, such that $d^{+}(v) \ge \frac{(n - \alpha )}{2 \alpha }$. $\square$

**Intuitive Ideas for the proof Lemma** 3.1. Let us begin by recalling that $(v_1, \ldots , v_n)$ is a *d*-out-degeneracy sequence of *D*. Also recall that, the aim of proving Lemma 3.1 is to be able to use it to bound the number of *k*-cuts in *D* of type-*i*. Consider any *k*-cut in *D* of type-*i*. By definition, $v_i \in L$ and for all $j>i$, $v_j \in R$. Thus, $v_i \in V_{\textrm{forceL}}$ and $\{v_j : j>i\} \subseteq V_{\textrm{forceR}}$. Thus, to prove Lemma 3.1, we essentially need to partition the vertices that appear before $v_i$ in $(v_1, \ldots ,v_n)$. Consider the non-neighbors of $v_i$. They induce a digraph whose independence number is strictly less than the independence number of *D*. Thus, they go to $V_{\textrm{induct}}$. Thus, we are now left with the goal of partitioning the set of neighbors of $v_i$ that appear before $v_i$ in $(v_1, \ldots ,v_n)$. Since $(v_1, \ldots , v_n)$ is a *d*-out-degeneracy sequence of *D*, the number of out-neighbors of $v_i$ that appear before $v_i$ in $(v_1, \ldots , v_n)$ is at most *d*. This set of neighbors goes to the set $V_{\textrm{small}}$. Finally, we are left with the set, say *X*, of vertices that appear before $v_i$ in $(v_1, \ldots , v_n)$ and are in-neighbors of $v_i$. Here, we observe that, if any vertex $v \in X$ has out-degree at least $k+1$ in *D*[*X*], then there are at least $k+1$ arc-disjoint paths from *v* to $v_i$ in $D[X \cup \{v_i\}]$, and hence in *D*. Thus, such a vertex *v* should always belong to same part as $v_i$ in any *k*-cut. Thus, such vertices goes to $V_{\textrm{forceL}}$. Finally, the remaining vertex set, say $X'$, has the property that each vertex in *X* has out-degree at most *k*. By Lemma 3.2, in such a case the size of $X'$ is at most $\alpha (2k+1)$, and hence $X'$ goes to $V_{\textrm{small}}$. We are now ready to prove Lemma 3.1 formally.

### Proof of Lemma 3.1

Let $(v_1, \ldots , v_n)$ be a *d*-out-degeneracy sequence of *D*. Consider the partition of *V*(*D*) into three parts: $\{v_i\}$, the predecessors of $v_i$ in this ordering, $V_P$ and the successors of $v_i$ in this ordering $V_S$. Formally, consider $V(D)=\{v_i\} \uplus V_P \uplus V_S$, where $V_P=\{v_j: j<i\}$ and $V_S= \{v_j:j>i\}$. Further consider the partition of $V_P$ into the set of vertices of $V_P$ that are neighbors of $v_i$, say $V_{P}^N$, and the set of vertices of $V_P$ that are non-neighbors of $v_i$, say $V_{P}^{NN}$. That is, $V(P) = V_{P}^N \uplus V_{P}^{NN}$. Next consider the partition of $V_{P}^N$ into two parts: $V_{P}^{ON}$ and $V_{P}^{IN}$ such that $V_{P}^{ON}$ is the set of vertices in $V_{P}^N$ that are out-neighbors of $v_i$ and $V_{P}^{IN}$ is the set of vertices in $V_{P}^N$ that are in-neighbors of $v_i$. Finally, consider the digraph induced on $V_{P}^{IN}$. We partition the set $V_{P}^{IN}$ based on the out-degree of the vertices in $D'=D[V_{P}^{IN} \cup \{v_i\}]$. We partition the set $V_{P}^{IN}$ into two parts: $V_{P,L}^{IN}$ and $V_{P,S}^{IN}$, in the following way. If $d^{+}_{D'}(v) \ge k+1$, $v \in V_{P,L}^{IN}$, otherwise $v \in V_{P,S}^{IN}$. Observe that, for each $v \in V_{P,S}^{IN}$, $d^{+}_{D''(v)} \le k$, where $D''=D[V_{P,S}^{IN} \cup \{v_i\}]$. We have the following from the above discussion.

$$\begin{aligned} V(D)&= \{v_i\} \uplus V_P \uplus V_S = \{v_i\} \uplus V_{P}^N \uplus V_{P}^{NN} \uplus V_S\\ {}&= \{v_i\} \uplus V_{P}^{ON} \uplus V_{P}^{IN} \uplus V_{P}^{NN} \uplus V_S\\&= \{v_i\} \uplus V_{P}^{ON} \uplus V_{P,L}^{IN} \uplus V_{P,S}^{IN} \uplus V_{P}^{NN} \uplus V_S. \end{aligned}$$

**Fig. 2:**
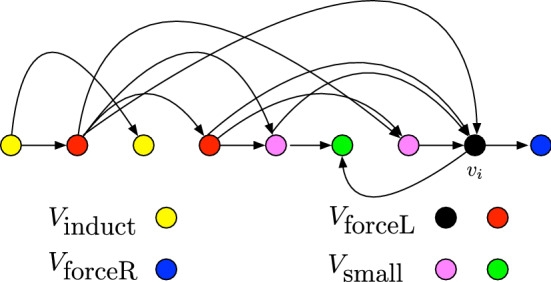
The vertex partition for the Sub-exponential FPT bound. Here the vertices are arranged in the linear order respecting the *d*-out-degeneracy sequence of *D*. Here $k=2$ and the partition of the vertices into the respective sets is demonstrated using appropriate colors (Color figure online)

We now claim that the desired partition $V(D) = V_{\textrm{induct}} \uplus V_{\textrm{forceL}} \uplus V_{\textrm{forceR}} \uplus V_{\textrm{small}}$ is such that, (1) $V_{\mathrm {\textrm{induct}}}=V_{P}^{NN}$, (2) $V_{\textrm{forceL}} = \{v_i\} \cup V_{P,L}^{IN}$, (3) $V_{\textrm{forceR}} = V_S$, and (4) $V_{\textrm{small}} = V_{P}^{ON} \cup V_{P,S}^{IN}$. An illustration depicting this partitioning can be found in Fig. 2. Let us now prove that the sets $V_{\mathrm {\textrm{induct}}}, V_{\textrm{forceL}}, V_{\textrm{forceR}}$ and $V_{\textrm{small}}$ satisfy the desired properties.

1. $V_{\mathrm {\textrm{induct}}}$: Observe that when $\alpha =1$, that is, when *D* is a tournament, $V_{P}^{NN}= \emptyset$. Therefore, in this case, $V_{\mathrm {\textrm{induct}}} = \emptyset$. Otherwise, since $D[V_{\mathrm {\textrm{induct}}}]$ is a subgraph of *D* and $D \in \mathcal {D}_{\alpha ,d}$, $D[V_{\mathrm {\textrm{induct}}}] \in \mathcal {D}_{\alpha ,d}$. Since $V_{\mathrm {\textrm{induct}}}$ only contains vertices that are non-neighbors of $v_i$, if $D[V_{\mathrm {\textrm{induct}}}]$ has an independent set, say *X*, of size $\alpha$ then $X \cup \{v_i\}$ is an independent set in *D* of size $\alpha +1$, which contradicts the fact that the size of any independent set in *D* is bounded by $\alpha$. Thus, $D[V_{\mathrm {\textrm{induct}}}] \in \mathcal {D}_{\alpha ',d}$, where $\alpha ' < \alpha$.
2. $V_{\textrm{forceL}}$: By the definition of a type-*i* cut, for any *k*-cut (*L*, *R*) of type-*i* in *D*, $v_i \in L$. We will now show that for any $v_j \in V_{P,L}^{IN}$, there exists $k+1$ arc-disjoint paths from $v_j$ to $v_i$. Thus, if (*L*, *R*) is a *k*-cut in *D* and $v_i \in L$, then for all $v_j \in V_{P,L}^{IN}$, $v_j \in L$. Consider any $v_j \in V_{P,L}^{IN}$. Recall that $d^{+}_{D'}(v_j) \ge k+1$ where $D'=D[V_{P}^{IN} \cup \{v_i\}]$ and $V_{P}^{IN}$ is the set of in-neighbors of $v_i$ in $V_P$. Consider the set of out-neighbours of $v_j$ in $D'$. Since the number of such out-neighbors is at least $k+1$ and each of these out-neighbors is an in-neighbor of $v_i$, we conclude that there are at least $k+1$ arc-disjoint paths from $v_j$ to $v_i$.
3. $V_{\textrm{forceR}}$: By the definition of type-*i* cut, $V_S \subseteq R$, for any type-*i* cut (*L*, *R*).
4. $V_{\textrm{small}}$: Since $(v_1,\ldots ,v_n)$ is a *d*-out-degeneracy sequence of *D*, $\vert V_{P}^{NO} \vert \le d$. We need to show that $\vert V_{P,S}^{IN} \vert \le \alpha (2k+1)$. Recall that, as observed before, for each $v \in V_{P,S}^{IN}$, $d^{+}_{D''}(v) \le k$, where $D''=D[V_{P,S}^{IN} \cup \{v_i\}]$. Since $D''$ is an induced subgraph of *D*, $D'' \in \mathcal {D}_{\alpha ,d}$. Also for each $v \in V(D'')$, $d^{+}_{D''}(v) \le k$. Thus, from Lemma 3.2, $\vert V(D'') \vert \le \alpha (2k+1)$. This proves that $\vert V_{P,S}^{IN} \vert \le \alpha (2k+1)$.

This concludes the proof. $\square$

## Sub-exponential FPT Algorithms for DFAS, Directed Cutwidth and OLA for Digraphs in $\mathcal {D}_{\alpha }$

In this section, we will give sub-exponential FPT algorithms for DFAS, Directed Cutwidth and Optimal Linear Arrangement when the input graph belongs to $\mathcal {D}_{\alpha }$, for some positive integer $\alpha$. All these algorithms are based on a three step procedure. The *first* is observing that the digraphs that are Yes-instances of these problems have *sub-exponential FPT* many *k*-cuts. The proofs for DFAS and Directed Cutwidth are based on showing that the digraph in the Yes-instances of the problems are *k*-out-degenerate, and hence, the bounds follow from Lemma 1.5. For OLA, we show that if there is an ordering of the vertex set of a digraph of cost at most *k* then the cutwidth of this digraph is $\mathcal {O}(k^{2/3})$. Hence, from the results for Directed Cutwidth, the number of *k*-cuts in the Yes-instances of OLA is also bounded. The *second* step is a procedure to enumerate all *k*-cuts of the input digraph. And the *third* is to do some dynamic programming procedure over these enumerated cuts to solve the respective problems. The last part of the algorithm (doing dynamic programming over *k*-cuts) is standard and is identical to the algorithm given by Fomin and Pilipczuk [13]. Proofs are given for completeness.

Before proceeding further, we make a small remark that the proofs of Lemmas 1.4 and 1.5 can be made constructive by using the constructive versions of the Gallai-Milgram’s Theorem, Chromatic Coding lemma and a polynomial time procedure to output a *d*-out-degeneracy sequence of a digraph. Thus, one can actually enumerate all the *k*-cuts in the input digraphs of these Lemmas using our algorithm. However, for the sake of completeness, we state in Lemma 4.1, a different procedure that using a standard branching, enumerates all the *k*-cuts in any digraph with polynomial delay.

### Lemma 4.1

(Lemma 7, [13]) *k*-cuts of a digraph *D* can be enumerated with polynomial-time delay.

### Sub-exponential Algorithm for Directed Feedback Arc Set

We begin by recalling the problem definition.
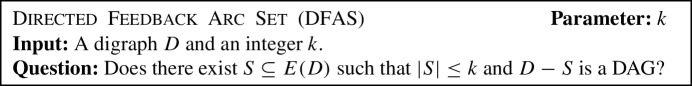


Such a set $S \subseteq E(D)$ is called a dfas of *D*. Observe that, a digraph *D* has a dfas of size at most *k* if and only if there exists an ordering, say $(v_1, \ldots ,v_n)$, of *V*(*D*) such that $\vert \sum _{i \in [n]} N^+(v_i) \cap \{v_j : j<i\} \vert \le k$, that is, the number of backward arcs in this ordering is at most *k*. Next we bound the number of *k*-cuts in the Yes-instances of DFAS.

#### Lemma 4.2

If (*D*, *k*) is a Yes-instance of DFAS and $D \in \mathcal {D}_{\alpha }$, then the number of *k*-cuts in *D* is at most $2^{c(\alpha +1)\sqrt{k} \log k} \cdot 2^ {2 \alpha (\alpha +1) \lceil \sqrt{k} \rceil \log ( (k (2 \alpha +1)+\alpha +1))} \cdot \log (k + \alpha (2k+1)) \cdot n^{\alpha +1}$, where *c* is a fixed absolute constant.

#### Proof

Since (*D*, *k*) is a Yes-instance of DFAS, there exists an ordering, say $(v_1, \ldots , v_n)$, of *V*(*D*), such that $\vert \sum _{i \in [n]} N^+(v_i) \cap \{v_j : j<i\} \vert \le k$. In particular, for any $i \in [n]$, $\vert N^+(v_i) \cap \{v_j : j<i\} \vert \le k$. Thus, $(v_1, \ldots ,v_n)$ is a *k*-out-degeneracy sequence of *V*(*D*). Therefore, the bound follows from Lemma 1.5. $\square$

Now we give the proof of Theorem 1.1.

#### Proof of Theorem 1.1

Using the algorithm of Lemma 4.1, we enumerate all *k*-cuts in *D*. If during the enumeration we exceed the bound given in Lemma 4.2, then we correctly conclude that (*D*, *k*) is a No-instance of DFAS. Otherwise, from Lemma 4.1, in time $2^{\mathcal {O}(\alpha ^2 \sqrt{k} \log (\alpha k))} \cdot n^{\mathcal {O}(\alpha )}$, we would have enumerated the set of all *k*-cuts in *D*. Let us denote this set by $\mathcal {C}$. We will solve the DFAS problem by doing a dynamic programming over the set $\mathcal {C}$ of *k*-cuts. Let *T* be the dynamic programming table indexed by a *k*-cut $(L,R) \in \mathcal {C}$ and an integer $i \in [k]$. For any $(L,R) \in \mathcal {C}$ and $i \in [k]$, we want *T*((*L*, *R*), *i*) to store the following information.

$$\begin{aligned} T((L,R),i) = {\left\{ \begin{array}{ll} 1 &{} \quad \text {if there exists an ordering } (v_1, \ldots , v_{\ell }) \text { of } L \\ {} &{} \quad \text {witnessing that } D[L] \text { has a dfas{} of size } i\text {, and } \\ {} &{} \quad (L {\setminus } \{v_{\ell }\}, R \cup \{v_{\ell }\} ) \in \mathcal {C} \\ 0 &{} \quad \text {otherwise } \end{array}\right. } \end{aligned}$$

Note that $T((V(D),\emptyset ),k) =1$ if and only if *D* has a dfas of size at most *k*. We now describe how we compute *T*((*L*, *R*), *i*), for any $(L,R) \in \mathcal {C}$ and $i \in [k]$. For all $i \in [k]$, $T((\emptyset , V(D)),i) = 1$. For any $(L,R) \in \mathcal {C}$, such that $L \ne \emptyset$, and any $i \in [k]$, $T((L,R),i) =1$ if and only if there exists $v \in L$ such that $(L {\setminus } \{v\}, R \cup \{v\}) \in \mathcal {C}$ and, if $\vert N^{+}_{L}(v) \vert =j$, then $T((L {\setminus } \{v\},R \cup \{v\}), i-j)=1$. Formally, $T((L,R),i) = \vee _{v \in L, (L {\setminus } \{v\}, R \cup \{v\}) \in \mathcal {C}} T((L {\setminus } \{v\}, R \cup \{v\}), i - |N^+_L(v)|)$. Thus, for any fixed *L*, *R*, *i*, *T*((*L*, *R*), *i*) can be computed in linear time by doing look-ups. Overall, *T*((*L*, *R*), *i*) can be computed in $|\mathcal {C}| n^{\mathcal {O}(1)}$ time.

We now prove that for any $(L,R) \in \mathcal {C}$ and $i \in [k]$, $T((L,R),i)=1$ if and only if there exists an ordering $(v_1, \ldots , v_{\ell })$ of *L* witnessing that *D*[*L*] has a dfas of size *i*, and $(L {\setminus } \{v_{\ell }\}, R \cup \{v_{\ell }\} ) \in \mathcal {C}$. We prove this by induction on $\vert L \vert$. When $\vert L \vert =0$, this is true because of the base case. By inductive hypothesis, assume that it holds for any $(L',R') \in \mathcal {C}$ such that $\vert L' \vert = \ell -1$, and for any $i \in [k]$. We will first prove that if $T((L,R),i)=1$, then there exists an ordering $(v_1, \ldots , v_{\ell })$ of *L* witnessing that *D*[*L*] has a dfas of size *i*, and $(L {\setminus } \{v_{\ell }\}, R \cup \{v_{\ell }\} ) \in \mathcal {C}$.

Since $T((L,R),i)=1$, there exists a vertex, say $v_{\ell } \in L$, such that $(L {\setminus } \{v_{\ell }\}, R \cup \{v_{\ell }\}) \in \mathcal {C}$ and if $\vert N^{+}_{L}(v_{\ell }) \vert =j$ then $T((L {\setminus } \{v_{\ell }\}, R \cup \{v_{\ell }\}),i-j) =1$. Since $T((L {\setminus } \{v_{\ell }\}, R \cup \{v_{\ell }\}),i-j) =1$, from induction hypothesis, $D[L {\setminus } \{v_{\ell }\}]$ has a dfas of size at most $i-j$. Let $(v_1, \ldots , v_{\ell -1})$ be the ordering of $L {\setminus } \{v_{\ell }\}$ witnessing this, that is, $\sum _{p \in [\ell -1]} \vert N^{+}(v_p) \cap \{v_q : q<p\} \vert \le i-j$. Since $\vert N^{+}_{L}(v_{\ell }) \vert =j$, $\sum _{p \in [\ell ]} \vert N^{+}(v_p) \cap \{v_q : q<p\} \vert \le i$. Thus, the ordering $(v_1, \ldots ,v_{\ell -1},v_{\ell })$ is a witness to the fact that *D*[*L*] has a dfas of size at most *i*.

We will now prove that if *D*[*L*] has a dfas of size at most *i* and $(v_1, \ldots , v_{\ell })$ is an ordering witnessing this such that $(L {\setminus } \{v_{\ell }\}, R \cup \{v_{\ell }\}) \in \mathcal {C}$, then $T((L,R),i)=1$. Clearly, if $\vert N^{+}(v_{\ell }) \vert =j$, then the ordering $(v_1, \ldots , v_{\ell -1})$ witnesses that $D[L {\setminus } \{v_{\ell }\}]$ has a dfas of size at most $i-j$. Thus, $T((L {\setminus } \{v_{\ell }\}, R \cup \{v_{\ell }\}),i-j)=1$. $\square$

### Sub-exponential Algorithm for Directed Cutwidth

Let *D* be a digraph. For an ordering $(v_1, \ldots , v_n)$ of *V*(*D*), the *width* of this ordering is $\max _{i \in [n-1]} \vert E(\{v_{i+1}, \ldots , v_n\}, \{v_1,\ldots , v_i\})\vert$. The *cutwidth* of *D*, denoted by ${\textbf {ctw}} (D)$, is the smallest possible width of an ordering of *V*(*D*).
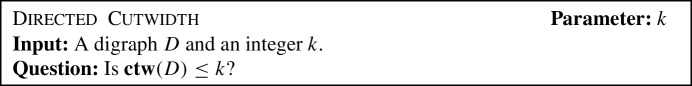


Next we bound the number of *k*-cuts in the Yes-instances of DFAS.

#### Lemma 4.3

If (*D*, *k*) is a Yes-instance of Directed Cutwidth and $D \in \mathcal {D}_{\alpha }$, then the number of *k*-cuts in *D* is at most $2^{c(\alpha +1)\sqrt{k} \log k} \cdot 2^ {2 \alpha (\alpha +1) \lceil \sqrt{k} \rceil \log ( (k (2 \alpha +1)+\alpha +1))} \cdot \log (k + \alpha (2k+1)) \cdot n^{\alpha +1}$, where *c* is a fixed absolute constant.

#### Proof

If (*D*, *k*) is a Yes-instance of DFAS, then there is an ordering, say $(v_1,\ldots , v_n)$, of *V*(*D*) of width at most *k*. Recall that, the width of an ordering $(v_1, \ldots , v_n)$ is $\max _{i\in [n-1]} \vert E(\{v_{i+1}, \ldots , v_n\}, \{v_1, \ldots , v_i\}) \vert$. Observe that if $\max _{i\in [n-1]} \vert E(\{v_1, \ldots , v_i\}, \{v_{i+1}, \ldots , v_n\}) \vert \le k$, then for each $i \in [n]$, $\vert N^{+}(v_i) \cap \{v_j : j <i\}\vert \le k$. Thus, *D* is *k*-out-degenerate. Thus, the bound follows from Lemma 1.5. $\square$

Now we give the proof of Theorem 1.2.

#### Proof of Theorem 1.2

Using the algorithm of Lemma 4.1, we enumerate all *k*-cuts in *D*. If during the enumeration we exceed the bound given in Lemma 4.3, then we correctly conclude that (*D*, *k*) is a No-instance of Directed Cutwidth. Otherwise, from Lemma 4.1, in time $2^{\mathcal {O}(\alpha ^2 \sqrt{k} \log (\alpha k))} \cdot n^{\mathcal {O}(\alpha )}$, we would have enumerated the set of all *k*-cuts in *D*. Let us denote this set by $\mathcal {C}$. We will solve the Directed Cutwidth problem by doing a dynamic programming over the set $\mathcal {C}$ of *k*-cuts. Let *T* be the dynamic programming table indexed by a *k*-cut $(L,R) \in \mathcal {C}$. For any $(L,R) \in \mathcal {C}$, we want *T*((*L*, *R*)) to store the following information.

$$\begin{aligned} T((L,R)) = {\left\{ \begin{array}{ll} 1 &{} \quad \text {if there exists an ordering of } L \text {, say } (v_1, \ldots , v_{\ell }) \text {,} \\ &{}\quad \text {such that for all } j \in [\ell -1], \\ {} &{}\quad \vert E(V(D) {\setminus } \{v_{1}, \ldots , v_j\}, \{v_1, \ldots , v_j\}) \vert \le k\\ 0 &{} \quad \text {otherwise } \end{array}\right. } \end{aligned}$$

Note that $T((V(D),\emptyset )) =1$ if and only if ${\textbf {ctw}} (D) \le k$. We now describe how we compute *T*((*L*, *R*)) for any $(L,R) \in \mathcal {C}$. Set $T((\emptyset , V(D))) = 1$. For any $(L,R) \in \mathcal {C}$ such that $L \ne \emptyset$, $T((L,R)) =1$ if and only if there exists $v \in L$ such that $(L {\setminus } \{v\}, R \cup \{v\}) \in \mathcal {C}$ and $T((L {\setminus } \{v\},R \cup \{v\}))=1$.

We now prove that for any $(L,R) \in \mathcal {C}$, $T((L,R))=1$ if and only if there exists an ordering of *L*, say $(v_1, \ldots , v_{\ell })$, such that for all $j \in [\ell -1]$, $\vert E(V(D) {\setminus } \{v_{1}, \ldots , v_j \}, \{v_1, \ldots , v_j\}) \vert \le k$. We prove this by induction on $\vert L \vert$. When $\vert L \vert =0$, this is true because of the base case. By inductive hypothesis, assume that for any $(L',R') \in \mathcal {C}$, such that $\vert L' \vert = \ell -1$, $T((L',R')) =1$ if and only if there exists an ordering of $L'$, say $(v_1, \ldots , v_{\ell -1})$, such that for all $j \in [\ell -2]$, $\vert E(V(D) {\setminus } \{v_{1}, \ldots , v_j\}, \{v_1, \ldots , v_j\}) \vert \le k$. Let $(L,R) \in \mathcal {C}$ be such that $\vert L \vert = \ell$. We will first prove that if $T((L,R))=1$, then there exists an ordering of *L*, say $(v_1, \ldots ,v_{\ell })$, such that for all $j \in [\ell -1]$, $\vert E(V(D) {\setminus } \{v_{1}, \ldots , v_j\}, \{v_1, \ldots , v_j\}) \vert \le k$. Since $T((L,R))=1$, there exists a vertex in *L*, say $v_{\ell }$, such that $(L {\setminus } \{v_{\ell }\}, R \cup \{v_{\ell }\})$ and $T((L {\setminus } \{v_{\ell }\},R \cup \{v_{\ell }\}))=1$. Since $T((L {\setminus } \{v_{\ell }\},R \cup \{v_{\ell }\}))=1$, from inductive hypothesis, there exists an ordering of $L {\setminus } \{v_{\ell }\}$, say $(v_1, \ldots ,v_{\ell -1})$, such that for all $j \in [\ell -2]$, $\vert E(\{v_{j+1}, \ldots ,v_n\}, \{v_1, \ldots ,v_j\}) \vert \le k$. Also, since $(L {\setminus } \{v_{\ell }\}, R \cup \{v_{\ell }\}) \in \mathcal {C}$, $\vert E(V(D) {\setminus } \{v_{1}, \ldots ,v_{\ell -1}\}, \{v_1, \ldots ,v_{\ell -1}\}) \vert \le k$. Thus, for the ordering $(v_1, \ldots ,v_{\ell })$ of *L*, for all $j \in [\ell -1]$, $\vert E(V(D) {\setminus } \{v_{1}, \ldots , v_j\}, \{v_1, \ldots , v_j\}) \vert \le k$.

We will now prove that if there exists an ordering of *L*, say $(v_1, \ldots ,v_{\ell })$, such that for all $j \in [\ell -1]$, $\vert E(V(D) {\setminus } \{v_{1}, \ldots , v_j\}, \{v_1, \ldots , v_j\}) \vert \le k$, then $T((L,R))=1$. Since $\vert E(V(D) {\setminus } \{v_{1}, \ldots , v_{\ell -1}\}, \{v_1, \ldots , v_{\ell -1}\}) \vert \le k$, $(L {\setminus } \{v_{\ell }\}, R \cup \{v_{\ell }\}) \in \mathcal {C}$. Also, since for all $j \in [\ell -2]$, $\vert E(V(D) {\setminus } \{v_{1}, \ldots , v_j\}, \{v_1, \ldots , v_j\}) \vert \le k$, therefore, $T((L{\setminus } \{v_{\ell }\}, R \cup \{v_{\ell }\}))=1$. Thus, $T((L,R))=1$. This concludes the proof. $\square$

### Sub-exponential Algorithm for Optimal Linear Arrangement

Let *D* be a digraph. For an ordering $\sigma =(v_1, \ldots , v_n)$ of *V*(*D*), the *cost* of $\sigma$ is

$$\begin{aligned} \sum _{(v_i,v_j) \in E(D)} (i-j) \cdot [i>j], \end{aligned}$$

that is, every arc directed backward in the ordering contributes a cost that is equal to the length of this arc, which is the distance between the end-points of this arc in the ordering. Recall that $[i >j]$, evaluates to 1 if $i >j$, to 0 otherwise.
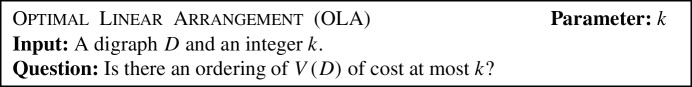


The following proposition gives an alternate definition of the cost of an ordering.

#### Proposition 3

[13] For a digraph *D* and an ordering $(v_1, \ldots , v_n)$ of *V*(*D*), the cost of this ordering is equal to $\sum _{i\in [n-1]} \vert E(\{v_{i+1},\ldots ,v_n\},\{v_1,\ldots ,v_i\})\vert$.

Lemma 4.4 shows a relation between the cost of an ordering and its width. Note that this lemma was already proved in [13], but the authors state the result for the case when the input digraph is a semi-complete digraph. We observe that the same proof works for any digraph. For the sake of completeness, we give the same proof here.

#### Lemma 4.4

For any digraph *D*, if there is an ordering say $(v_1,\ldots ,v_n)$ of *V*(*D*), of cost at most *k*, then ${\textbf {ctw}} (D) \le (2k)^{\frac{2}{3}}$.

#### Proof

Since $(v_1, \ldots , v_n)$ is an ordering of cost at most *k*, from Proposition 3, $\sum _{i\in [n-1]} \vert E(\{v_{i+1},\ldots ,v_n\}, \{v_1,\ldots ,v_i\})\vert \le k$. Fix an arbitrary $i \in [n-1]$. We will show that $\vert E(\{v_{i+1}, \ldots , v_n\}, \{v_1,\ldots ,v_i\}) \vert \le (2k)^{\frac{2}{3}}$. Let $\vert E(\{v_{i+1}, \ldots , v_n\}, \{v_1,\ldots ,v_i\}) \vert =\ell$. For any arc $(v_q,v_p) \in E(D)$, such that $p <q$, the length of the arc $(v_q,v_p)$ is equal to $q-p$. Observe that, for any *r*, the number of arcs of length exactly *r* with tail in $\{v_{i+1}, \ldots , v_n\}$ and head in $\{v_1, \ldots ,v_i\}$ is at most *r*. Thus, for any *r*, the total number of arcs of length at most *r*, with tail in $\{v_{i+1}, \ldots ,v_n\}$ and head in $\{v_1, \ldots ,v_i\}$, is at most $\frac{r(r+1)}{2}$. In particular, the number of arcs of length at most $\sqrt{\ell }-1$, with tail in $\{v_{i+1}, \ldots ,v_n\}$ and head in $\{v_1, \ldots ,v_i\}$ is at most $\frac{\sqrt{\ell }(\sqrt{\ell } -1)}{2} \le \frac{\ell }{2}$. Since $\vert E( \{v_{i+1}, \ldots , v_n\}, \{v_1,\ldots ,v_i\}) \vert =\ell$, the number of arcs of length at least $\sqrt{\ell }$ with tail in $\{v_{i+1}, \ldots , v_n\}$ and head in $\{v_1 , \ldots , v_i\}$ is at least $\frac{\ell }{2}$. Since $\sum _{i\in [n-1]} \vert E(\{v_{i+1},\ldots ,v_n\}, \{v_1,\ldots ,v_i\})\vert \le k$, we have that $k \ge \sqrt{\ell } \cdot \frac{\ell }{2}$. Thus, $\ell \le (2k)^{\frac{2}{3}}$. Thus, we have shown that for any arbitrary $i \in [n-1]$, $\vert E(\{v_{i+1}, \ldots , v_n\}, \{v_1,\ldots ,v_i\}) \vert \le (2k)^{\frac{2}{3}}$. Thus, $(v_1, \ldots , v_n)$ is an ordering of cutwidth at most $(2k)^{\frac{2}{3}}$. $\square$

Next we bound the number of *k*-cuts in the Yes-instances of OLA.

#### Lemma 4.5

If (*D*, *k*) is a Yes-instance of OLA and $D \in \mathcal {D}_{\alpha }$, then the number of *k*-cuts in *D* is at most $2^{c(\alpha +1)k^{\frac{1}{3}} \log k} \cdot 2^ {2 \alpha (\alpha +1) \lceil k^{\frac{1}{3}} \rceil \log ( (k (2 \alpha +1)+\alpha +1))} \cdot \log (k + \alpha (2k+1)) \cdot n^{\alpha +1}$, where *c* is a fixed absolute constant.

#### Proof

Since *D* is a Yes-instance of OLA, from Lemma 4.4, ${\textbf {ctw}} (D) \le (2k)^{\frac{2}{3}}$. Thus, $(D, (2k)^{\frac{2}{3}})$ is a Yes-instance of Directed Cutwidth. Hence, from Lemma 4.3, the number of *k*-cuts in *D* are bounded by the desired function. $\square$

#### Proof of Theorem 1.3

Using the algorithm of Lemma 4.1, we enumerate all *k*-cuts in *D*. If during the enumeration we exceed the bound given in Lemma 4.5, then we correctly conclude that (*D*, *k*) is a No-instance of OLA. Otherwise, from Lemma 4.1, in time $2^{\mathcal {O}(\alpha ^2 k^{\frac{1}{3}} \log (\alpha k))} \cdot n^{\mathcal {O}(\alpha )}$, we would have enumerated the set of all *k*-cuts in *D*. Let us denote this set by $\mathcal {C}$. We will solve OLA by doing a dynamic programming over the set $\mathcal {C}$ of *k*-cuts. Let *T* be the dynamic programming table indexed by a *k*-cut $(L,R) \in \mathcal {C}$ and an integer $i \in [k]$. For any $(L,R) \in \mathcal {C}$ and $i \in [k]$, we want *T*((*L*, *R*), *i*) to store the following information.

$$\begin{aligned} T((L,R),i) = {\left\{ \begin{array}{ll} 1 &{} \quad \text {if there exists an ordering of } L \text {, say } (v_1, \ldots ,v_{\ell }) \text {,} \\ {} &{} \quad \text{ such } \text{ that } \sum _{j \in [\ell ]} \vert E(V(D) {\setminus } \{v_{1}, \ldots ,v_j\}, \{v_1, \ldots , v_j\}) \vert \le i\\ 0 &{} \quad \text {otherwise } \end{array}\right. } \end{aligned}$$

Note that $T((V(D),\emptyset ),k) =1$ if and only if *D* has an ordering of cost at most *k*. We now describe how we compute *T*((*L*, *R*), *i*) for any $(L,R) \in \mathcal {C}$ and $i \in [k]$. For all $i \in [k]$, $T((\emptyset , V(D)),i) = 1$. For any $(L,R) \in \mathcal {C}$ such that $L \ne \emptyset$, and any $i \in [k]$, $T((L,R)) =1$ if and only if there exists $v \in L$ such that $(L {\setminus } \{v_{\ell }\}, R \cup \{v_{\ell }\})$ and $T((L {\setminus } \{v\},R \cup \{v\}), i-j)=1$, where $j = \vert E(R,L) \vert$.

We now prove that for any $(L,R) \in \mathcal {C}$ and integer $i \in [k]$, $T((L,R),i)=1$ if and only if there exists an ordering of *L*, say $(v_1, \ldots ,v_{\ell })$, such that $\sum _{j \in [\ell ]} \vert E(V(D) {\setminus } \{v_{1}, \ldots ,v_j\}, \{v_1, \ldots , v_j\}) \vert \le i$. We prove this by induction on $\vert L \vert$. When $\vert L \vert =0$, this is true because of the base case. By inductive hypothesis, assume that for any $(L',R') \in \mathcal {C}$ such that $\vert L' \vert = \ell -1$, and for any $p \in [k]$, $T((L',R'),p) =1$ if and only if there exists an ordering of *L*, say $(v_1, \ldots ,v_{\ell })$, such that $\sum _{j \in [\ell ]} \vert E(V(D) {\setminus } \{v_{1}, \ldots ,v_j\}, \{v_1, \ldots , v_j\}) \vert \le i$. Let $(L,R) \in \mathcal {C}$ be such that $\vert L \vert = \ell$ and $i \in [k]$. We will first prove that if $T((L,R),i)=1$, then there exists an ordering of *L*, say $(v_1, \ldots ,v_{\ell })$, such that $\sum _{j \in [\ell ]} \vert E(V(D) {\setminus } \{v_{1}, \ldots ,v_j\}, \{v_1, \ldots , v_j\}) \vert \le i$. Let $j = \vert E(R,L)\vert$. Since $T((L,R),i)=1$, there exists a vertex in *L*, say $v_{\ell }$, such that $(L {\setminus } \{v_{\ell }\}, R \cup \{v_{\ell }\}) \in \mathcal {C}$ and $T((L {\setminus } \{v_{\ell }\}, R \cup \{v_{\ell }\} ), i -j)=1$. From inductive hypothesis, there exists an ordering of $L {\setminus } \{v_{\ell }\}$, say $(v_1, \ldots , v_{\ell -1})$, such that $\sum _{p \in [\ell -1]} \vert E(V(D) {\setminus } \{v_{1}, \ldots ,v_p\}, \{v_1, \ldots , v_p\}) \vert \le i-j$. Since $j = \vert E(R,L)\vert$, for the ordering $(v_1, \ldots ,v_{\ell })$ of *L*, $\sum _{p \in [\ell ]} \vert E(V(D) {\setminus } \{v_{1}, \ldots ,v_p\}, \{v_1, \ldots , v_p\}) \vert \le i$.

We will now prove that if there exists an ordering of *L*, say $(v_1, \ldots ,v_{\ell })$, such that $\sum _{j \in [\ell ]} \vert E(V(D) {\setminus } \{v_{1}, \ldots ,v_j\}, \{v_1, \ldots , v_j\}) \vert \le i$, then $T((L,R),i)=1$. Observe from the definition of this ordering $(v_1, \ldots , v_{\ell })$ that $(L {\setminus } \{v_{\ell }\}, R \cup \{v_{\ell }\})$ is an *i*-cut in *D*. Since $i \le k$, $(L {\setminus } \{v_{\ell }\}, R \cup \{v_{\ell }\}) \in \mathcal {C}$. Clearly, if $\vert E(R,L) \vert =j$, then $\sum _{p \in [\ell -1]} \vert E(V(D) {\setminus } \{v_{1}, \ldots ,v_p\}, \{v_1, \ldots , v_{p}\}) \vert \le i-j$. Thus, $T((L {\setminus } \{v_{\ell }\}, R \cup \{v_{\ell }\}),i-j)=1$ implying that $T((L,R),i)=1$. This concludes the proof. $\square$

## Conclusion

In this paper, we designed sub-exponential time parameterized algorithms for DFAS, Directed Cutwidth and OLA on digraphs of bounded independence number. We thus significantly generalized known results for the restricted case of input digraphs that are tournaments. Towards this, we obtained an upper bound on the number of *k*-cuts in digraphs in ${\mathcal {D}}_\alpha$. This bound is our main contribution, which we believe to find further implications in the future, and to be of independent interest. We conclude with an open problem: Do DFAS, Directed Cutwidth and OLA admit polynomial kernels on ${\mathcal {D}}_\alpha$?
